# Determinants of soluble angiotensin-converting enzyme 2 concentrations in adult patients with complex congenital heart disease

**DOI:** 10.1007/s00392-020-01782-y

**Published:** 2020-12-05

**Authors:** Tanja Raedle-Hurst, Sarah Wissing, Nils Mackenstein, Rima Obeid, Juergen Geisel, Stefan Wagenpfeil, Hashim Abdul-Khaliq

**Affiliations:** 1 Department of Pediatric Cardiology, Saarland University Medical Center, Homburg/Saar, Germany; 2 Department of Clinical Chemistry and Laboratory Medicine, Saarland University Medical Center, Homburg/Saar, Germany; 3 Institute of Medical Biometry, Epidemiology and Medical Informatics, Saarland University Medical Center, Homburg/Saar, Germany

**Keywords:** Soluble ACE2, Complex congenital heart disease, Heart failure, COVID-19

## Abstract

**Background:**

Angiotensin-converting enzyme (ACE) 2 is known to be a functional receptor for SARS-CoV-2 in the current pandemic. Soluble ACE2 (sACE2) concentrations are elevated in patients with various cardiovascular disorders including heart failure.

**Methods:**

In a total of 182 consecutive adult patients with complex congenital heart disease (CHD) and 63 healthy controls, sACE2 concentrations were measured in serum using the Human ACE2^®^ assay by Cloud-Clone Corporation and associated with clinical, laboratory and echocardiographic parameters.

**Results:**

Median sACE2 levels were increased in patients with complex CHD as compared to healthy controls (761.9 pg/ml vs 365.2 pg/ml, *p* < 0.001). Moreover, sACE2 concentrations were significantly elevated in patients with a higher NYHA class ≥ III (1856.2 pg/ml vs 714.5 pg/ml in patients with NYHA class I/II, *p* < 0.001). Using linear regression analysis, higher sACE2 levels were associated with a higher NYHA class ≥ III, more severe CHD, a morphological left systemic ventricle, higher creatinine and the use of mineralocorticoid receptor antagonists (MRA) in the univariable model. The use of ACE inhibitors or angiotensin receptor blockers (ARB) was associated with lower sACE2 levels. In the multivariable model, higher sACE2 levels were independently associated with a higher NYHA class ≥ III (*p* = 0.002) and lower sACE2 levels with the use of ACE inhibitors or ARB (*p* = 0.001).

**Conclusion:**

Soluble ACE2 concentrations were significantly increased in all types of complex CHD with highest levels found in patients with NYHA class ≥ III. Moreover, a higher NYHA class ≥ III was the most significant determinant that was independently associated with elevated sACE2 concentrations.

**Graphic abstract:**

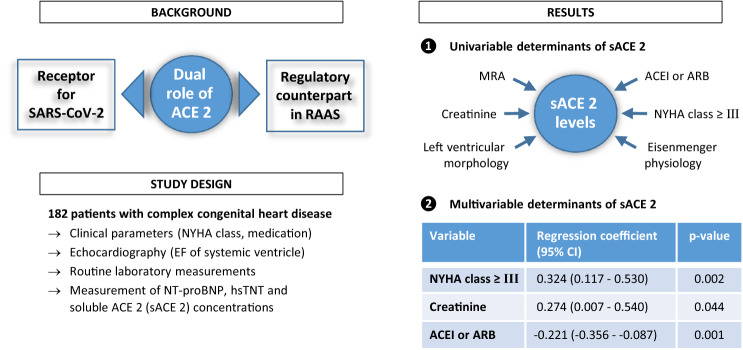

## Introduction

Angiotensin-converting enzyme 2 (ACE2) is a membrane-bound carboxymonopeptidase that converts Angiotensin I and Angiotensin II into Angiotensin 1–9 and Angiotensin 1–7, respectively, and is highly expressed on the surface of different organ cells in the heart, lungs, kidneys and testis [1–3]. The soluble form of ACE2 (by proteolytic shedding of membrane-bound ACE2) is also a key component of the counter-regulatory pathway within the renin–angiotensin–aldosterone system (RAAS) which plays a crucial role in the regulation of blood pressure, heart failure, diabetes and chronic kidney disease [4–6].

The current coronavirus SARS-CoV-2 uses ACE2 as a cellular receptor to invade target cells. In particular, the spike protein of SARS-CoV-2 is processed by transmembrane protease-serine 2 and favors the binding of the spike protein to ACE2 to enter the host cells [6]. To date, an increasing number of publications have confirmed the role of ACE2 receptor for the cell entry mechanism of SARS-CoV-2 in the current pandemic [2, 3, 6]. Several epidemiological studies have also shown the severity of the SARS-CoV-2 disease (COVID-19) in older patients and particularly those with cardiovascular rather than pulmonary predisposing risk factors [7–9]. Thus, sACE2 concentrations were found to be elevated in men and older patients with different cardiovascular morbidities, such as hypertension, diabetes, coronary heart disease and heart failure [8, 10–14]. These interactions of ACE2 may explain the higher vulnerability and predisposition of such patients to have a severe or fatal outcome after SARS-CoV-2 infection [15–17].

Adult patients with complex congenital heart disease (CHD) may also represent a vulnerable group of patients due to comorbidities, residual heart defects with longstanding pressure or volume overload as well as reduced intrinsic myocardial function finally resulting in symptomatic heart failure in early or mid-term adulthood [18]. Data on the SARS-CoV-2 infection rate in patients with CHD are still lacking. According to data from different countries and the Johns Hopkins University, children and young people seem to have a less severe time course and fatality rate with COVID-19 than older adults [19, 20] what may be due to lower sACE2 levels [21]. Moreover, an increased vulnerability of older and male patients with cardiovascular disease or heart failure can be seen that could also be related to increased sACE2 levels [10–12, 22]. In adult patients with complex CHD who often present with altered myocardial function or even symptomatic heart failure, data on sACE2 levels are lacking. Thus, in the light of the dual role of ACE2 during the current pandemic, the aim of our study was to assess sACE2 levels in this particular patient cohort and to evaluate determinants of sACE2 concentrations with special emphasis on factors that are independently associated with increased sACE2 concentrations.

## Methods

### Patients

In our outpatient clinic for adults with CHD, several prospective studies for the evaluation of different biomarkers in serum as well as micro-RNA signatures in whole blood had been performed in the past [23–25]. To assess sACE2 concentrations, existing serum samples of these patients frozen at -80° C representing the pre-COVID-19 era were used for analysis. Thus, serum samples of 182 consecutive patients with complex CHD ≥ 18 years of age who were regularly seen in our outpatient clinic between 22/01/2015 and 02/12/2019 were analyzed in the present study. Complex CHD was classified according to current ACC/AHA guidelines [26] and mainly related to severe congenital heart defects including all forms of pulmonary atresia, cyanotic CHD, Eisenmenger syndrome, transposition of the great arteries (TGA), common arterial trunk, all kinds of single ventricle physiology with or without Fontan palliation as well as CHD at increased risk for heart failure, such as tetralogy of Fallot [27]. Patients with mild or moderate CHD, pregnancy, severe renal dysfunction or dialysis and incapability to understand or sign informed consent were excluded. Of the 182 patients enrolled in the study, 60/182 (33.0%) patients had corrective surgery of congenital right heart disease (CRHD), such as pulmonary atresia, tetralogy of Fallot or common arterial trunk, 62/182 (34.1%) patients had TGA of whom 40/62 (64.5%) patients had a systemic morphological right ventricle (TGA-RV) and 22/62 (35.5%) patients a systemic morphological left ventricle (TGA-LV) after arterial switch operation, 37/182 (20.3%) patients showed a single ventricle physiology after complete Fontan palliation (FONT) and in 23/182 (12.6%) patients a non-corrected cyanotic heart defect or Eisenmenger syndrome classified as Eisenmenger physiology (EIS) was present. Mean age was 30.1 ± 10.5 years (range 18–62 years) and 103 patients were male (56.6%). The study protocol has been described in detail previously [23]. 63 healthy subjects with a mean age of 31.6 ± 11.7 years (range 18–61 years) served as controls. The control group was mainly recruited within our institution and consisted of medical students, medical staff or family members. To rule out a hemodynamically relevant heart abnormality, a physical examination and two-dimensional echocardiography were performed in all healthy participants. Clinical data of controls and patients are given in Table 1. All patients and healthy controls gave written informed consent before enrollment.

**Table 1:** Comparison of healthy controls and patients

Variables	Healthy controls (*n* = 63)	All patients (*n* = 182)	*p* value*
Age at enrollment (years)	31.6 ± 11.7	30.1 ± 10.5	0.474
Male sex	39/63 (61.9%)	103/182 (56.6%)	0.462
Body mass index (kg/m^2^)	23.2 ± 3.3	23.9 ± 4.8	0.579
NYHA class	1.0 ± 0	1.5 ± 0.7	< 0.001
Systolic blood pressure (mmHg)	120.8 ± 10.9	123.3 ± 13.9	0.304
Diastolic blood pressure (mmHg)	70.2 ± 7.7	70.8 ± 9.4	0.829
Transcutaneous oxygen saturation at rest (%)	98.2 ± 1.2	95.0 ± 5.9	< 0.001
Ejection fraction of SV (%)	62.4 ± 4.4	54.9 ± 9.1	< 0.001
Enddiastolic volume of SV (ml)	107.9 ± 26.9	131.9 ± 62.2	0.006
Endsystolic volume of SV (ml)	40.5 ± 12.6	61.9 ± 37.7	< 0.001
VTI above aortic valve (cm)	27.4 ± 4.3	24.4 ± 4.5	< 0.001
sACE2 concentrations (pg/ml)	365.2 (237.7–656.3)	761.9 (419.2–1277.7)	< 0.001

Mean ± standard deviation or median (interquartile interval) are used

*NYHA* New York Heart Association, *SV* systemic ventricle, *VTI* velocity time integral, *sACE2* soluble angiotensin-converting enzyme 2

^*^Mann–Whitney *U* test

### Data and biochemical analyses

Clinical data of the patients were collected from medical records. Echocardiographic data sets were analysed on an Echopac server (Echopac Version 6, GE Healthcare, Horten, Norway) and assessed by investigators blinded to the laboratory results.

Venous blood samples were drawn into standard sampling tubes shortly after echocardiography, centrifuged at 3000*g* and serum removed, allocated and frozen at − 80 °C before analysis of ACE2 levels. In the patient group, routine laboratory tests, such as liver function tests and creatinine measurements, were performed using standard laboratory techniques. NT-proBNP and high sensitive troponin T levels were measured using an electrochemiluminescence sandwich immunoassay (Cobas^®^ proBNP II and troponin T hs STAT, Roche Diagnostics, Basel, Switzerland) on the Elecsys^®^ 2010 analyzer. Levels of ACE2 were measured using a commercially available sandwich enzyme immunoassay (Human ACE2^®^, Cloud-Clone Corporation, Houston, Texas, USA) on the FLUOstar^®^ Omega Microplate Reader (BMG LABTECH Inc., Cary, North Carolina, USA) and has been described in detail previously [28]. All biochemical analyses were performed by investigators blinded to the clinical and echocardiographic data of the patients. Glomerular filtration rate (GFR) was estimated using the chronic kidney disease epidemiology collaboration (CKD-EPI) creatinine equation [29].

### Statistical analysis

Data were analysed using standard statistical software (SPSS version 25; SPSS Inc., Chicago, Illinois). Continuous variables are expressed as mean ± standard deviation or median (interquartile interval) as appropriate. Differences between unpaired groups were analysed using a Mann–Whitney *U* or Kruskal–Wallis test for continuous variables and a chi-square test for nominal variables. Linear regression analysis was used in a univariable and multivariable way to identify determinants of sACE2 levels. For regression analysis, sACE2 concentrations were log_10_ transformed due to the skewed distribution. In the multivariable model, variables were entered that gave statistically significant values in the univariable model and did not show any multicollinearity. The multivariable model was based on forward stepwise regression. A two-tailed *p* value < 0.05 was considered statistically significant.

## Results

### Study population and sACE2 levels

**Fig. 1:**
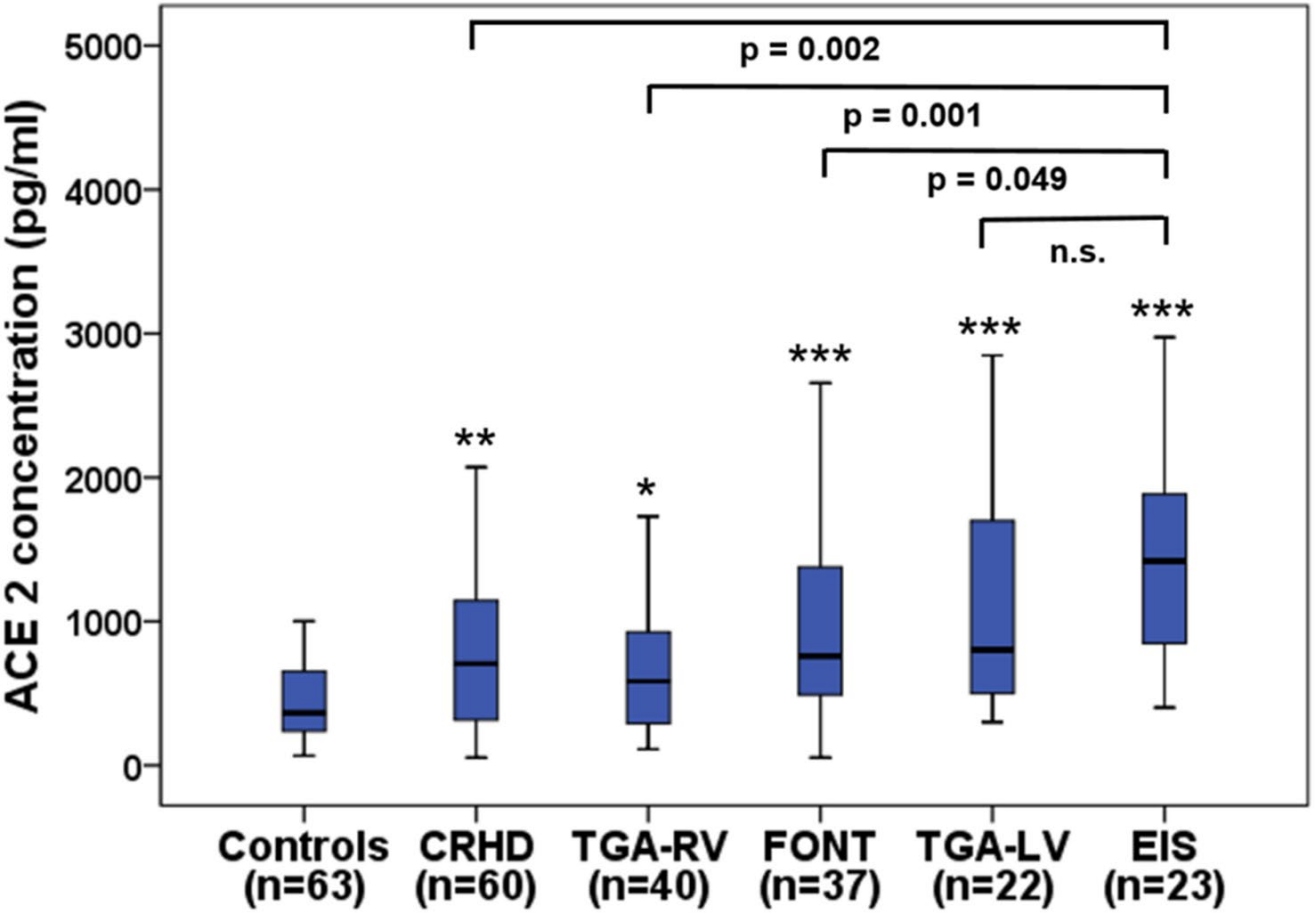
Boxplots displaying sACE2 levels in healthy controls and patients with various types of congenital heart defects. *p < 0.05, **p = 0.001, ***p < 0.001 as compared to controls. *CRHD* corrected congenital right heart disease, *TGA* transposition of the great arteries, *RV* systemic right ventricle, *FONT* Fontan palliation, *LV* systemic left ventricle, *EIS* Eisenmenger physiology

**Fig. 2:**
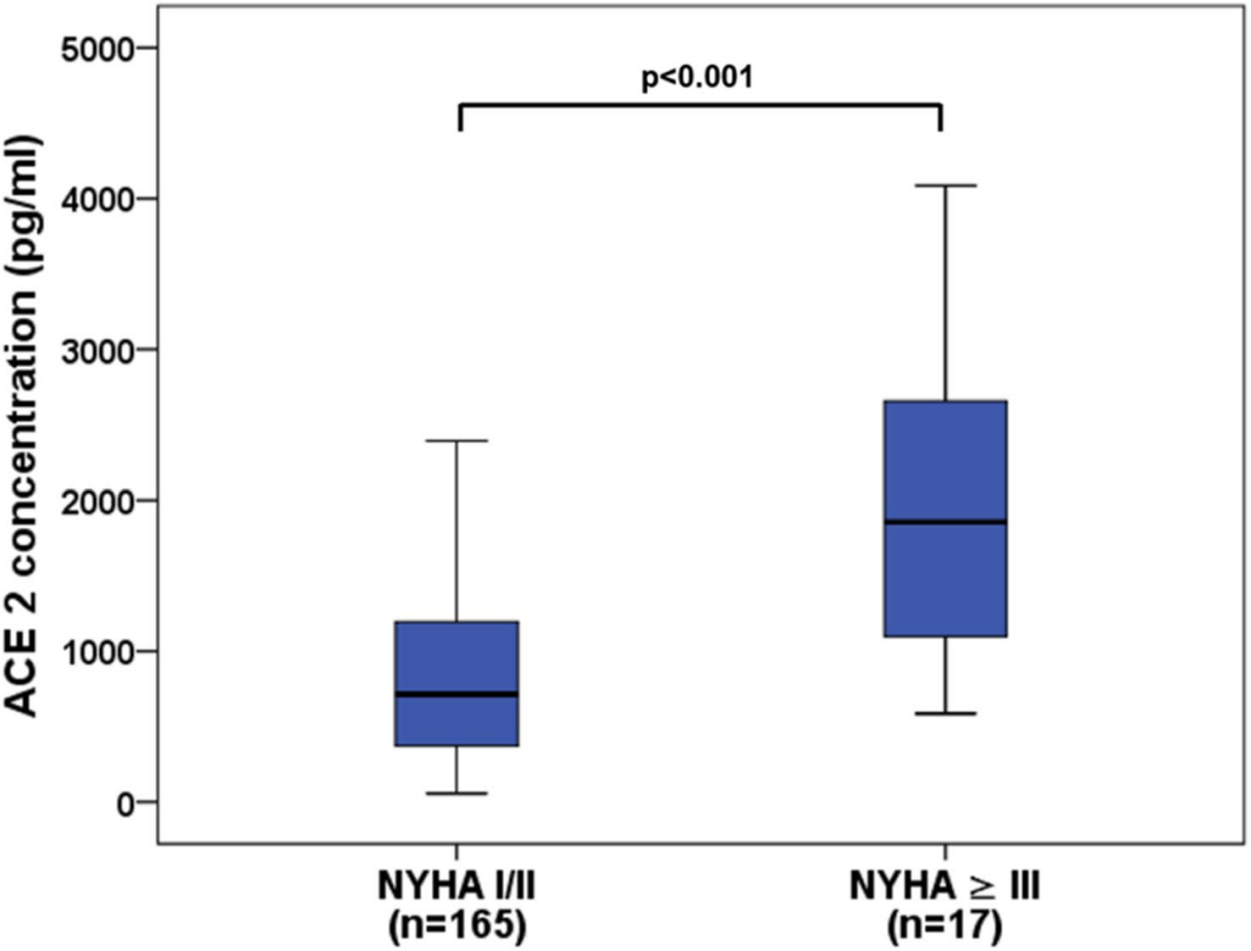
Boxplots illustrating sACE2 levels in patients with NYHA class I/II compared to NYHA class ≥ III

Clinical characteristics of patients based on quartile serum sACE2 concentrations are shown in Table 2. In patients with complex CHD, sACE2 levels ranged from 55.0 to 4526.1 pg/ml with a median of 761.9 pg/ml (419.2–1277.7 pg/ml) and were significantly elevated as compared to healthy controls showing a median sACE2 level of 365.2 pg/ml (237.7–656.3 pg/ml; *p* < 0.001). Significantly elevated sACE2 levels were found in all subgroups of CHD patients in comparison to the healthy control group with highest levels seen in patients with Eisenmenger physiology (median 1418.4 pg/ml) (Fig. 1). Moreover, patients with a systemic morphological left ventricle displayed higher sACE2 levels than those with a systemic morphological right ventricle (median 841.7 pg/ml vs 633.9 pg/ml, *p* = 0.026). Soluble ACE2 levels were also significantly increased in patients with a higher NYHA class ≥ III reflecting advanced heart failure as compared to patients with NYHA class I/II (median 1856.2 pg/ml vs 714.5 pg/ml, *p* < 0.001) (Fig. 2).

**Table 2:** Baseline characteristics of patients according to sACE2 quartiles

Variables	Quartile 1 (< 419.2) (*n* = 45)	Quartile 2 (419.2–761.9) (*n* = 46)	Quartile 3 (761.9–1277.7) (*n* = 46)	Quartile 4 (> 1277.7) (*n* = 45)	*p* value*
Age at enrollment (years)	30.4 ± 9.8	29.5 ± 10.1	29.2 ± 10.2	31.4 ± 12.0	0.757
Male sex	25/45 (55.6%)	25/46 (54.3%)	28/46 (60.9%)	25/45 (55.6%)	0.925
NYHA class	1.4 ± 0.5	1.4 ± 0.5	1.5 ± 0.7	1.8 ± 0.8	0.090
Systolic blood pressure (mmHg)	122.9 ± 12.0	125.1 ± 14.8	121.6 ± 13.9	123.6 ± 15.0	0.849
Diastolic blood pressure (mmHg)	69.6 ± 7.6	72.4 ± 9.8	69.7 ± 9.3	71.6 ± 10.7	0.489
Transcutaneous oxygen saturation at rest (%)	96.8 ± 2.8	96.3 ± 6.2	94.8 ± 5.8	92.2 ± 8.2	0.012
Presence of atrial fibrillation	0/45 (0%)	1/46 (2.2%)	2/46 (4.3%)	3/45 (6.7%)	0.326
Left ventricular morphology of SV	25/45 (55.6%)	31/46 (67.4%)	39/46 (84.8%)	33/45 (73.3%)	0.160
Ejection fraction of SV (%)	55.2 ± 8.5	56.5 ± 8.1	55.2 ± 9.4	53.0 ± 10.3	0.326
VTI above aortic valve (cm)	24.4 ± 3.9	25.8 ± 4.8	24.0 ± 4.7	23.6 ± 4.2	0.200
Creatinine (mg/dl)	0.81 (0.75–0.95)	0.85 (0.71–0.96)	0.85 (0.75–0.95)	0.94 (0.81–1.09)	0.013
Estimated GFR (ml/min)	106.9 (96.5–117.6)	109.5 (96.3–121.8)	109.5 (91.3–120.2)	99.6 (71.7–118.5)	0.313
NT-proBNP (ng/l)	151.0 (64.5–236.3)	206.4 (87.8–314.9)	125.5 (67.0–237.2)	255.3 (89.6–627.7)	0.022
High sensitive troponin T (pg/ml)	5.0 (3.0–6.0)	4.0 (3.0–8.0)	5.0 (3.0–7.0)	5.0 (3.0–13.5)	0.496
Medication					
ACEI or ARB	14/45 (31.1%)	8/46 (17.4%)	9/46 (19.6%)	5/45 (11.1%)	0.116
β-blockers	13/45 (28.9%)	10/46 (21.7%)	15/46 (32.6%)	17/45 (37.8%)	0.402
MRA	3/45 (6.7%)	7/46 (15.2%)	9/46 (19.6%)	11/45 (24.4%)	0.135
Loop diuretics or thiazides	10/45 (22.2%)	9/46 (19.6%)	11/46 (23.9%)	18/45 (40.0%)	0.116
Antiarrhythmics	2/45 (4.4%)	0/46 (0%)	3/46 (6.5%)	7/45 (15.6%)	0.072

Mean ± standard deviation or median (interquartile interval) are used

*sACE2* soluble angiotensin-converting enzyme 2, *NYHA* New York Heart Association, *SV* systemic ventricle, *VTI* velocity time integral, *GFR* glomerular filtration rate, *ACEI* angiotensin-converting enzyme inhibitors, *ARB* angiotensin receptor blockers, *MRA* mineralocorticoid receptor antagonists

^*^Kruskal–Wallis test

### Association of sACE2 levels with different variables

Linear regression analysis revealed that higher sACE2 levels were significantly correlated with a higher NYHA class ≥ III, the presence of Eisenmenger physiology representing more severe CHD, a left ventricular morphology of the systemic ventricle, higher creatinine levels as well as the use of mineralocorticoid receptor antagonists (MRA) in the univariable model (Table 3). In contrast, the use of ACE inhibitors (ACEI) or angiotensin receptor blockers (ARB) was associated with lower sACE2 levels. Interestingly, sACE2 concentrations were only weakly related to NT-proBNP (*r* = 0.127, *p* = 0.024) or high sensitive troponin T levels (*r* = 0.274, *p* = 0.010) in our study cohort.

**Table 3:** Univariable and multivariable linear regression analysis for determinants of sACE2 levels

	Univariable analysis		Multivariable analysis	
	Regression coefficient	*p* value	Regression coefficient (95% CI)	*p* value
Age at enrollment	− 0.001	0.835	–	–
Male sex	0.018	0.756	–	–
NYHA class ≥ III	0.414	< 0.001	0.324 (0.117–0.530)	0.002
Diagnosis of EIS	0.313	< 0.001	–	–
Presence of atrial fibrillation	0.301	0.065	–	–
Left ventricular morphology of SV	0.138	0.028	–	–
Transcutaneous oxygen saturation at rest	− 0.019	< 0.001	–	–
Creatinine	0.436	0.001	0.274 (0.007–0.540)	0.044
Glomerular filtration rate	− 0.003	0.027	–	–
Medication				
ACEI or ARB	− 0.202	0.006	− 0.221 (− 0.356–0.087)	0.001
β-blockers	0.023	0.713	–	–
MRA	0.167	0.034	–	–
Loop diuretics or thiazides	0.121	0.067	–	–
Antiarrhythmics	0.219	0.053	–	–

*sACE2* soluble angiotensin-converting enzyme 2, *NYHA* New York Heart Association, *EIS* Eisenmenger physiology, *ACEI* ACE inhibitors, *ARB* angiotensin receptor blockers, *MRA* mineralocorticoid receptor antagonists

In the multivariable model, however, the most significant factor independently associated with higher levels of sACE2 was found to be a higher NYHA class ≥ III (*p* = 0.002) whereas the use of ACEI or ARB was the most significant factor associated with lower levels of sACE2 (*p* = 0.001).

## Discussion

In our study, significantly higher sACE2 serum levels were found in adult patients with complex CHD in comparison to healthy controls indicating the relationship of ACE2 expression to cardiovascular disease. Soluble ACE2 levels differed significantly between the studied CHD subgroups with highest concentrations found in the EIS subgroup, thus reflecting the severity of CHD (Fig. 1). Interestingly, patients with TGA-LV after the arterial switch procedure showed higher sACE2 levels than those with TGA-RV after the atrial switch operation what may be due to the morphology of the systemic ventricle since patients with a systemic morphological right ventricle showed lower sACE2 levels than those with a systemic morphological left ventricle. In addition, sACE2 concentrations were significantly increased in patients with NYHA class ≥ III reflecting advanced heart failure (Fig. 2).

### Soluble ACE2 levels in other patient cohorts

Our results are consistent with a previous study illustrating increasing plasma sACE2 concentrations according to NYHA class in heart failure patients with highest levels found in the acute setting [30]. Moreover, in that study, sACE2 concentrations were found to be elevated in patients receiving MRA or loop diuretics whereas no difference was seen with the use of ACEI or ARB medication. Although brain natriuretic peptide levels were significantly related to sACE2 levels in that study, correlation of NT-proBNP with sACE2 was rather weak in our cohort of patients.

Elevated sACE2 levels were also detected in male and female patients with coronary heart disease using the same sACE2 assay by Cloud-Clone Corporation as in our study [31]. Surprisingly, the coronary heart disease patients displayed markedly higher sACE2 concentrations (about 5000–6000 pg/ml) than our CHD patients with NYHA class ≥ III (approximately 2000 pg/ml). These differences may be due to the different cardiovascular comorbidities (e.g. higher prevalence of cardiovascular risk factors, such as arterial hypertension or diabetes) and the ischemic pathophysiology of altered myocardial function in the cohort with coronary heart disease. Hence, sACE2 levels were also significantly higher in patients with multi-vessel than single-vessel disease in that study. In contrast, altered myocardial function in CHD patients is mainly due to longstanding pressure or volume overload.

### Determinants of sACE2 levels

In the linear regression analysis, increased sACE2 levels were significantly related to a higher NYHA class ≥ III, more severe CHD, left ventricular morphology of the systemic ventricle, higher creatinine values and the use of MRA in the univariable model (Table 2). However, multivariable analysis revealed that a higher NYHA class ≥ III was the most significant determinant that was independently associated with higher sACE2 concentrations. In addition, the use of ACEI or ARB medication was independently associated with lower sACE2 concentrations. Our results are in accordance with a previous study investigating two independent cohorts of patients with left heart failure illustrating that ACEI or  ARB uses were independent predictors of lower sACE2 concentrations whereas the use of MRA was an independent predictor of higher sACE2 concentrations [22]. In contrast to that study, however, sACE2 levels were not found to be higher in male or older patients in our study population what may be due to the different age of the studied patients. In our CHD cohort, patients were rather young with a mean age of about 30 years in contrast to the older age of the patients studied by Sama et al. with a median age of 70 years [22].

### Study limitations

Our study had some limitations that need to be addressed. First, evaluation of the direct association of the measured sACE2 concentrations and the infection risk or severity of COVID-19 disease is currently not possible because data on the incidence of COVID-19 disease is not yet available for our study cohort. Moreover, sACE2 concentrations may not necessarily represent membrane-bound ACE2 expression because previous studies indicate that ACE2 shedding is mediated by a disintegrin and metalloproteinase (ADAM)-17 and may not be applied to the total extent of tissue-bound ACE2 activity [32–34]. Thus, the relation of sACE2 and the extent of ADAM-17 activity or membrane-bound ACE2 shedding is still unclear. Nevertheless, in the clinical setting, male sex, older age, the presence of cardiovascular risk factors or heart failure are  known to be highly predictive for a severe course of COVID-19 disease [8, 16] and could be due to increased sACE2 concentrations [21, 22, 35]. However, further studies are warranted to evaluate the direct relationship of sACE2 concentrations and the susceptibility or course of COVID-19 disease in adult patients with complex CHD.

## Conclusion

Soluble ACE2 concentrations were significantly elevated in adults with various types of complex CHD as compared to healthy controls with highest levels found in those with Eisenmenger physiology or a higher NYHA-class ≥ III reflecting advanced heart failure. However, a higher NYHA class ≥ III was the most significant determinant that was independently associated with increased sACE2 levels.
